# Quantifying the Effectiveness of Environmental Regulations on Green Total Factor Productivity: Evidence Based on China’s Environmental Protection Interview Program

**DOI:** 10.3390/ijerph20042980

**Published:** 2023-02-08

**Authors:** Dan Pan, Yi Yu, Fanbin Kong

**Affiliations:** 1 School of Economics, Jiangxi University of Finance and Economics, Nanchang 330013, China; 2 School of Economic Management, Nanjing Forestry University, Nanjing 210037, China

**Keywords:** environmental regulation, green economic development, environmental protection interview, green total factor productivity, difference-in-differences method

## Abstract

The effectiveness of environmental regulations on green total factor productivity (GTFP) is controversial, and the mechanisms of the relationship between environmental regulation and GTFP are unknown. In this article, we take advantage of the Environmental Protection Interview (EPI) program—the harshest environmental monitoring program in Chinese history—to carry out a natural experiment to estimate the effect of environmental regulation on GTFP. Applying a time-varying difference-in-differences model based on city panel data from 2003 to 2018 in China, we determined that the EPI can lead to an average GTFP promotion of 35.6%, but the effect of the EPI is not consistent in the long term. A heterogeneity analysis documented that the effect of the EPI on GTFP is more significant in cities with low initial GTFP levels and low economic levels. A mechanism analysis showed that the EPI increases GTFP, basically, through technical creativity and industrial structure upgrading.

## 1. Introduction

Since the 18th Party Congress in 2012, China has attached unprecedented importance to green economic development—an advanced type of economic development that incorporates environmental protection and economic development [1]. The Chinese government also committed to establishing peak carbon emissions by 2030 and achieving carbon neutrality by 2060. Well-designed environmental regulation arrangements are prerequisites to achieving these goals. However, the effectiveness of the environmental regulation policy in China has been declining due to local governments’ weak implementation [2]. This is because the primary aim of local governments in China is to pursue economic growth rather than sustainable development; thus, it is quite difficult for local officials to reach an effective consensus on environmental governance [3]. Therefore, the intensification of the central government’s administration of local governments to better implement environmental policies is a key part of China’s environmental management efforts. In this context, in 2014, the Chinese central government initiated the Environmental Protection Interview (EPI) program to address the gap in the implementation of environmental policies, aiming to foster green economic development [4]. As a new type of environmental enforcement supervision, EPI is regarded as a major transition: it is the harshest environmental monitoring program in Chinese history [5].

The existing literature has confirmed the role of the EPI in environmental management, and documented that the EPI can effectively decrease air pollution [6], water pollution [7], and other types of pollution [8]. However, the role of the EPI—or, broadly, environmental regulation—in economic development, especially green economic development, is controversial. There are two opposing theories that clarify the influence of environmental regulation on green economic development. One theory is the “Porter hypothesis”, which holds that reasonable environmental regulation can realize the win–win ambition of environmental protection and economic development [9]. The other theory is the “compliance costs theory”, which argues that rigid environmental governance policies raise the costs of pollution emissions treatment, thus crowding out productive investment and reducing the capacity of green economic growth. Each of these two theories is supported by considerable literature [10,11,12]. Most of the studies support the Porter hypothesis [4,13,14]. In addition, although the EPI has been found to have a beneficial impact on economic growth in China, little knowledge is available about the mechanisms behind its effectiveness.

Our paper adds to the relevant literature in three respects. First, to our knowledge, this paper is the first to provide a rigorous quantitative estimation of the effectiveness of the EPI on GTFP. Since the EPI is mainly used by the central government to press local governments to implement their responsibilities for environmental protection in response to serious environmental problems, existing works are mainly concerned with investigating the effect of the EPI on environmental quality from an ecological perspective; little literature exists on estimating the economic impact of the EPI. In this study, we take advantage of the EPI to carry out a quasi-natural experiment. We employ a difference-in-differences (DID) method to measure the EPI’s influence on green economic development. Green total factor productivity (GTFP)—a comprehensive indicator that indicates the level of economic development of a country or a region by considering economic growth, resource-saving measures, and environmental preservation in a broad manner [15]—is used to measure the green economic development. We provide evidence that the EPI has a positive impact on green economic development, adding information to the continuing debate on the effectiveness of the EPI on green economic development and providing valuable recommendations for future decisions on environmental regulation in China.

Second, we propose a theoretical framework and quantitatively analyze the mechanisms related to the effectiveness of the EPI on GTFP. Previous studies regarding the effectiveness of the EPI mostly lacked an empirical investigation of these mechanisms. The “Porter hypothesis” and the “compliance costs theory” only provide theoretical explanations, not empirical tests, for these mechanisms. In this article, we consider the mechanisms of the relationship between the EPI and GTFP. We verify that technical creativity and industrial structure upgrading are two of the main mechanisms. Our findings are useful additions to the literature on the EPI and its regulation effects and provide empirical evidence for optimizing the EPI and environmental regulation in other fields.

Third, compared with existing research on the economic impact of environmental regulation, which is mainly focused on developed countries such as the United States and some European countries [16,17], we investigate the effects of environmental regulation on green economic development in China, the world’s largest developing country. In developing countries, large numbers of people experience great harm from pollution, while continuing to rely, economically, on highly polluting manufacturing industries [18,19]. However, little is known about the environmental and economic impacts of environmental regulation in developing countries, despite the significant policy developments [19]. Our findings highlight the enormous environmental and economic benefits of environmental regulation in China. These benefits are extremely important for global environmental legislation, particularly in developing countries with similar economic systems that must choose between economic growth and environmental sustainability.

This framework is organized as follows. Section 2 reviews the literature and introduces China’s EPI. Section 3 documents the theoretical framework. Section 4 describes the data and method used. Section 5 provides the empirical estimation results, which include the baseline results, a series of robustness tests, and the heterogeneity analysis. Section 6 further explores the mechanism analysis of the EPI’s influence on GTFP. Section 7 concludes our argument and provides policy recommendations.

## 2. Literature Review and Institutional Background

### 2.1. Literature Review

As a major renovation in the history of environmental regulation, the EPI’s effectiveness has drawn the attention of academics. Numerous scholars have explored the validity of the EPI on environmental pollution management from an ecological perspective, having reached a consensus on the conclusion that the EPI can improve environmental quality. For instance, Jin et al. [6] concluded that the EPI can have a short-term policy benefit in terms of air quality enhancement. Zhao et al. [7] documented that EPI implementation can improve air quality and water quality, but the policy effect cannot be sustainable in the long term. Chen and Zhou [8] claimed that the EPI might also have a short-term effect in terms of various pollution. Pan et al. [20] found that the EPI can reduce water pollution with a long-term policy effect.

Economic performance is also a factor that cannot be ignored by governments in the formulation of the EPI. However, little literature explores the economic performance of the EPI, and their arguments are controversial. For example, Lv et al. [13] argued that the EPI promotes economic performance in high-pollution areas. Yu [4] found that the EPI accelerated the green innovation of the enterprise to promote economic development. Pan et al. [20] further claimed that the EPI is economically cost-effective since it can generate a total amount of CNY 520.97 billion in health benefits to society without hurting economic development. However, Zhou and Shen [14] documented that the EPI inhibits enterprises’ technical creativity, which in turn decreases economic development.

Through reviewing the above literature, we found that the existing literature mainly considers the EPI’s effect on environmental quality. Few studies have empirically explored the effectiveness of the EPI on green economic development; their outcomes are controversial, which hinds our understanding of the true economic performance of the EPI. To overcome these shortcomings, this paper attempts to use a natural experiment approach to empirically evaluate the EPI’s effect on green economic growth. We also attempt to disclose the mechanism of the EPI on green economic growth, which thus assists us in obtaining a precise understanding of the EPI’s operating mechanism.

### 2.2. Institutional Background: EPI in China

China’s environmental governance is contradictory: the central government’s intense focus on environmental protection conflicts with the local governments’ inadequate enforcement of environmental policies [21]. This is because local officials in China prefer economic development over environmental sustainability under the motivation of promotion, which is inconsistent with the central governments’ environmental legislative objectives [22].

To overcome the dilemma of local environmental governance failure, the EPI generates widespread interest and becomes a positive force in environmental management. The EPI is frequently utilized in China as a form of negotiation between administrative subjects and their counterparts [6]. In 2014, the Ministry of Environmental Protection (MEP) in China launched the official “environmental supervision” process [7]. Additionally, in 2020, the Ministry of Ecology and Environment issued the “Interview Measures of the Ministry of the Ecological Environment”, which provided an institutional basis for the EPI system. The EPI breaks the inefficiency of environmental regulation enforcement through the oversight of the local governments instead of the oversight of enterprises. The EPI process, which begins with inviting provincial environmental protection bureaus and city officials by the central government, can exert the full influence of authority transmission and authority supervision in China’s bureaucratic system and intensify pressure on local government offices and related departments, with an end result that local governments will attach importance to environmental protection work from top to bottom. For instance, the environmental quality decreases dramatically and has greatly aroused negative social impacts, with more public complaints about environmental management. Under the EPI’s surveillance, the cities interviewed are forced to accomplish environmental protection goals through enhancing supervision or enforcing legislation implementation. In general, the government can consolidate resources promptly, impose severe penalties, and provide rigorous accountability mechanisms to incentivize officials to tackle environmental problems through the implementation of the EPI [20].

**Figure 1:**
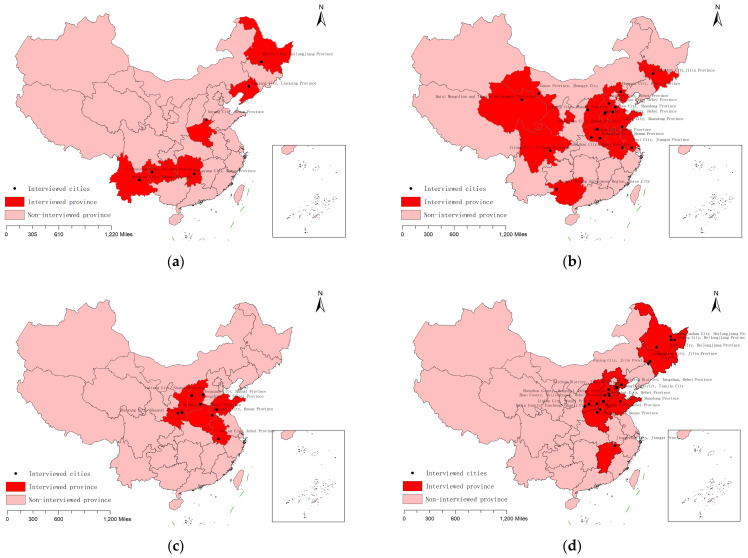

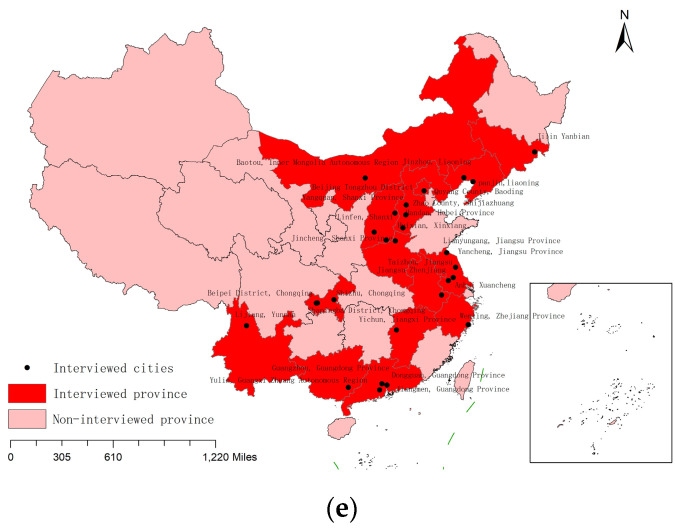
The geographical distribution of China’s EPI cities from 2014 to 2018. (**a**) The distribution of the EPI cities in 2014. (**b**) The distribution of the EPI cities in 2015. (**c**) The distribution of EPI cities in 2016. (**d**) The distribution of EPI cities in 2017. (**e**) The distribution of EPI cities in 2018. **Source:** This data was collated by the authors themselves and the sources are as follows: http://www.gov.cn/xinwen/2019-01/07/content_5355429.htm; http://www.gov.cn/xinwen/2016-12/07/content_5144822.htm (accessed on 30 January 2023).

Figure 1 shows the distribution of EPI cities from 2014 to 2018 in China. In detail, six provinces were interviewed in 2014. As the implementation of the EPI continued, the number of EPI cities was increased, with twelve provinces and eighteen cities in 2015, five provinces and eight cities in 2016, nine provinces and twenty-three cities in 2017, and fourteen provinces and twenty-seven cities in 2018.

## 3. Theoretical Framework

### 3.1. The Impact of the EPI on GTFP

As we stated above, two opposing theories clarify the impact of environmental regulation on green economic development. One is the “Porter hypothesis”, proving that environmental regulation has a positive impact on green economic development. The other is “compliance costs theory”, which argues that environmental regulation will inhibit green economic development. Regarding the EPI, based on prior analyses and the specific policies implemented in the EPI [4,13,14], we tend to support the idea of Porter hypothesis that the EPI can promote GTFP. The reasons are as follows. First, under the pressure of the EPI, the local governments will implement more effective measures to curb the release of pollutants and thus can promote GTFP. The EPI is an administrative measure to interview the accountability of local governments that are unable to undertake their environmental protection responsibilities. After the EPI, the MEP will urge local governments to bear prime duties for environmental management [6] and will set specific requirements and time limits for the local officials to rectify the environmental problems [14]. The MEP will expose them to public supervision if they fail to address environmental issues within the agreed period; even the local officials’ promotions would be affected by their performance with respect to the EPI [20]. This pressure may encourage local governments to take real action to address environmental issues, which is beneficial for improving GTFP. For example, after being interviewed, the responsible comrades of Puyang city, Henan province, China, said that they would consider this interview a warning and that they would succeed in environmental supervision and rectification to reverse the passive situation (http://news.sohu.com/a/525644411_121106991 (accessed on 30 January 2023). The Yiyang county of Jiangxi Province in China committed to giving priority to the prevention and control of environmental protection (https://www.sohu.com/a/518995950_121106994 (accessed on 30 January 2023).

Second, the EPI will compel enterprises to actively upgrade industrial structure and stimulate green innovation, which can advance the GTFP [23]. Under the pressure of the EPI, the local government will strengthen its oversight of the enterprises’ production activities to reduce pollution within a limited time [24]. For example, the local government will impose high penalties on or even stop the operation of polluting industries [25]. Some EPI cities only permit the establishment of environmentally friendly industries, such as energy-saving and environmental protection industries, to move in [8]. Through these measures, the enterprises, especially the polluting enterprises, will pay more attention to clean production to avoid fines and forced closures [26].

Hence, we bring the forward hypothesis:

**Hypothesis 1.**  *The EPI can significantly promote GTFP.*

### 3.2. The Mechanisms of the EPI on GTFP

**Figure 2:**
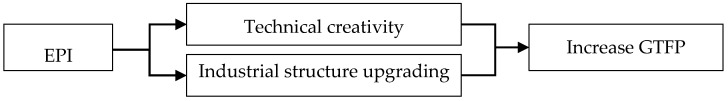
The mechanism of the EPI on GTFP.

What is the mechanism through which the EPI promotes GTFP? Based on previous research, we propose that the EPI can boost GTFP through two mechanisms: the technical creativity effect and the industrial structure upgrading effect (see Figure 2).

First, the EPI will force the enterprises, especially those that contribute to pollution, to adopt environmentally friendly technical creativity and thereby have a positive effect on GTFP. Technical creativity is the fundamental motivation of green development [27]. Following the “Porter hypothesis”, proper environmental legislation can fully motivate the enterprises’ productivity innovation, enhance production efficiency, and compensate for environmental regulation compliance costs [9]. The EPI, as a strict environmental regulation tool in China, can drive firms to engage in technical creativity toward green economic development [28]. For instance, under the EPI, the government will force the polluting enterprises to undergo green transformation by shutting down or rectifying high-pollution factories [29]. The government will also impose heavy environmental fines on companies for their high pollution emissions, which urges the enterprises to implement green technical creativity [23].

Second, the EPI can urge the local government to upgrade its industrial structure and thus foster GTFP promotion. The EPI, as an appropriate environmental regulation, can stimulate the upgrade of industrial structure [30]. As we stated above, under the pressure of the EPI, a direct way for local governments to decrease environmental pollution is to decrease the ratio of highly polluting industries and increase the ratio of energy-saving and environmentally friendly industries [31]. In this way, the industrial structure will be shift in a more environmentally friendly direction and is beneficial to GTFP promotion [32]. Hence, we bring the forward hypothesis:

**Hypothesis 2.**  *EPI can significantly improve GTFP through technical creativity.*

**Hypothesis 3.**  *EPI can significantly improve GTFP through industrial structure upgrading.*

## 4. Methodology and Data

### 4.1. Method

We treat EPI’s implementation as a quasi-natural experiment, and we evaluate whether EPI can promote GTFP by using DID method. DID method is diffusely used to estimate the policy effect, which can deal with the endogenous problems caused by omitted variables [33]. Considering the difference in EPI’s implementation time in various cities, this paper employs the time-varying DID model according to Beck et al. [34]. The formula is shown below:

(1) $${GTFP}_{it}=\alpha_{0}+\alpha_{1}{EPI}_{it}+\alpha_{2}X_{it}+\delta_{i}+\theta_{t}+\varepsilon_{it}$$

where ${GTFP}_{it}$ denotes GTFP in city i at year t. $\alpha_{0}$ and ${EPI}_{it}$ represent the constant term and EPI dummy variable, respectively. ${EPI}_{it}=1$ represents if city i implemented EPI at year t, otherwise 0. $\alpha_{1}$ represents the coefficient of ${EPI}_{it}$ on GTFP. $X_{it}$ denotes the control variables influencing GTFP. $\delta_{i}$, $\theta_{t}$, and $\varepsilon_{it}$ denotes regional fixed effect, time fixed effect, and the random error term, respectively. Based on our hypothesis 1 that EPI can promote GTFP, the coefficient of $\alpha_{1}$ is positive.

Equation (1) determines the average effects of the EPI on GTFP. To examine the dynamic effects of the EPI on GTFP, this paper established the following dynamic effect model by referring to Zhang et al. [35]:

(2) $${GTFP}_{it}=\pi_{0}+\sum_{k=0}^{k=4}\beta_{k}{EPI}_{it}^{k}+\beta_{5}X_{it}+\delta_{i}+\theta_{t}+\varepsilon_{it}$$

The dummy variable ${EPI}_{it}^{k}=1$ represents the post-EPI city, otherwise ${EPI}_{it}^{k}=0$.$\pi_{0}$ represents the constant term. $\beta_{k}$ is the coefficient of ${EPI}_{it}^{k}$, representing the impact of the EPI after the implementation of the EPI in year k. The other variables are the same as Equation (1).

### 4.2. Data and Variables

To examine the impact of the EPI on GTFP, we collect data on GTFP, EPI data, and other variables influencing EPI’s implementation. Our final sample consists of unbalanced panel data of 4272 observations of 267 cities from 2003 to 2018. The reason for only 267 cities as our research sample is that some cities (such as Chaohu, Sansha, Bijie, Laiwu, etc.) from the study sample are excluded owing to serious missing data in these cities.

#### 4.2.1. Dependent Variable: GTFP

The dependent variable is GTFP. We use the Global-Malmquist-Luenberger index (GML) calculated by the directional distance function (DDF) to measure GTFP [36]. GML is a widely used tool to represent GTFP [37,38]. We use the Stata software to calculate the GML index based on DDF in 267 Chinese cities. Specifically, the calculation of GTFP is as follows:

Firstly, we assumed that there are n decision units (DMU), each of which utilizes k inputs ($x=\left(x_{1},\cdots ,x_{k}\right)\in R_{k}^{+}$) to obtain j desired outputs $y=\left(y_{1},\cdots ,y_{j}\right)\in R_{j}^{+}$ and m undesired outputs ($b=\left(b_{1},\cdots ,b_{m}\right)\in R_{m}^{+}$). And then we constructed the production possibility set of the current period (t), which includes both the desired output and undesired output.

(3) $$P^{t}\left(x^{t}\right)=\left\{\left(y^{t},b^{t}\right)|x^{t}\to \left(y^{t},b^{t}\right),t=1,2,\cdots ,T\right\}$$

where $x^{t}$, $y^{t}$, and $b^{t}$ represent the vector of the input variables, the desired output, and undesired output in current period t, respectively.

Secondly, we built up the DDF of the current period which is presented below:

(4) $$D^{t}\left(x^{t},y^{t},b^{t};g\right)=sup\left\{\zeta |(y^{t}+\zeta g_{y},b^{t}-\zeta g_{b})\in P^{t}\left(x^{t}\right)\right\}$$

where $g=\left(g_{y}-g_{b}\right)$ denotes the directivity vector, ζ is the DDF used for maximizing the desired output and minimizing the undesired output.

Thirdly, we formulated the global production possibilities set which is the concatenation of all current production possibilities sets.

(5) $$P^{G}\left(x\right)=P^{1}\left(x^{1}\right)\cup P^{2}\left(x^{2}\right)\cup \cdots {\cup P}^{T}\left(x^{T}\right)$$

Then, we set up the global DDF as below:

(6) $$D^{G}\left(x,y,b\right)=sup\left\{\zeta |(y+\zeta y,b-\zeta b)\in P^{G}\right\}$$

Finally, we constructed a GML index based on global DDF. And we also provide the solution of the four DDFs in the GML index function by using the DEA linear programming model. We take current period t as an example, the current period DDF ($D^{t}\left(x^{t},y^{t},b^{t}\right)$) and the global DDF ($D^{G}\left(x^{t},y^{t},b^{t}\right)$) built on the set of global production possibilities ($P^{G}$) can be obtained by solving the following two linear programs, respectively. Similarly, we can accordingly obtain the t+1 period DDF ($D^{t+1}\left(x^{t+1},y^{t+1},b^{t+1}\right)$) and the global DDF ($D^{G}\left(x^{t+1},y^{t+1},b^{t+1}\right)$). The details are as follows:

(7) $${GML}_{t+1}^{t}\left(x^{t},y^{t},b^{t},x^{t+1},y^{t+1},b^{t+1}\right)=\frac{1+D^{G}\left(x^{t},y^{t},b^{t}\right)}{1+D^{G}\left(x^{t+1},y^{t+1},b^{t+1}\right)}\;=\frac{1+D^{t}\left(x^{t},y^{t},b^{t}\right)}{1+D^{t+1}\left(x^{t+1},y^{t+1},b^{t+1}\right)}\times \left[\frac{1+D^{G}\left(x^{t},y^{t},b^{t}\right)}{1+D^{t}\left(x^{t},y^{t},b^{t}\right)}\times \frac{1+D^{t+1}\left(x^{t+1},y^{t+1},b^{t+1}\right)}{1+D^{G}\left(x^{t+1},y^{t+1},b^{t+1}\right)}\right]={EC}_{t+1}^{t}\times {TC}_{t+1}^{t}\;D^{t}\left(x^{t},y^{t},b^{t}\right)=\max\zeta {;D}^{G}\left(x^{t},y^{t},b^{t}\right)=\max\zeta \;s.t.\left\{\begin{array}{c} y^{t}z^{t}\geq (1+\zeta )y_{l}^{t}\\ b^{t}z^{t}=(1-\zeta )b_{l}^{t}\\ x^{t}z^{t}\leq x_{l}^{t};z^{t}\geq 0 \end{array}\right.\;s.t.\left\{\begin{array}{c} \sum_{1}^{T}y^{t}z^{t}\geq (1+\zeta )y_{l}^{t}\\ \sum_{1}^{T}b^{t}z^{t}=(1-\zeta )b_{l}^{t}\\ \sum_{1}^{T}x^{t}z^{t}\leq x_{l}^{t};z^{t}\geq 0 \end{array}\right.$$

where ${GML}_{t+1}^{t}$, ${EC}_{t+1}^{t}$, and ${TC}_{t+1}^{t}$ are the input-output efficiency, the change of technical efficiency, and the technical progress of the DMU from two periods, respectively. $z^{t}$ is the weight coefficient vector of the evaluated unit in the effective decision unit combination. $x_{l}^{t}$, $y_{l}^{t}$, and $b_{l}^{t}$ are the factor input, desired output, and undesired output values of the lth decision unit, respectively.

If ${GML}_{t+1}^{t}$, ${EC}_{t+1}^{t}$, and ${TC}_{t+1}^{t}$ are greater than 1, they represent the improvement of the input-output efficiency, technical efficiency, and technical progress, respectively. If ${GML}_{t+1}^{t}$, ${EC}_{t+1}^{t}$, and ${TC}_{t+1}^{t}$ are less than 1, they represent the reduction of input-output efficiency, technical efficiency, and technical retrogression, respectively. Therefore, through the analysis of the GML index, we can observe the changing trends of GTFP and the change of influencing factors, to provide more accurate improvement schemes for GTFP utilization in various cities.

However, GML can simply capture the changing trend in GTFP, but not the magnitude of GTFP. We followed the study of Zhong et al. [39] to obtain the GTFP in each city by setting the GTFP of each city in the base year of 2003 to 1 and multiplying it cumulatively. For example, suppose that all GTFPs in 2003 are 1, then, the GTFP in 2004 would be the GTFP in 2003 multiplied by the GML index: ${GTFP}_{2004}={GTFP}_{2003}\times {GML}_{2003–2004}$. The GTFP of other years can be calculated similarly.

There are three input indicators (Labor, Capital, and energy) and two output indicators (desired output and undesired output) used to calculate GTFP. Labor is determined by the sum of employees per city at the year-end [40]. Capital is valued using the perpetual inventory method, referring to Xia and Xu [41], setting the depreciation rate at 5%, and then calculating the capital stock for each city with 2003 as the base period. Energy is measured by total electricity consumption as a proxy variable according to Feng et al. [42] and Yan et al. [43].

In terms of output indicators, the expected output is denoted by the actual Gross Domestic Product (GDP) through using the GDP price index to deflate nominal GDP to eliminate the influence of price factors [44]. The undesired output is the composite pollution index synthesized from three pollutants, including industrial wastewater release, SO2 discharge, and industrial fumes emission [33]. We employ the entropy value method to estimate the composite pollution index.

#### 4.2.2. Independent Variable: EPI Dummy

The independent variable is EPI. When a city falls within the interviewed cities, the EPI dummy variable equals 1; otherwise, 0. From 2014 to 2018, the specific number of cities included in the treatment group was 6, 15, 7, 13, and 13, respectively, totaling of 54 cities.

#### 4.2.3. Control Variables: Five Other Variables

We control for five control variables to reflect the affective factors of GTFP: population density, industrial structure, transportation level, fiscal intervention, and foreign direct investment.

(1) Population density. We use the total population as a percentage of administrative area to measure this variable. The impact of population density on GTFP is ambiguous. For one thing, it may promote GTFP since reasonable population density can have economies of scale and thus can increase resource use efficiency and promote GTFP within a certain range [45]. For the other thing, it may also restrain GTFP. This is because regions with high population density may experience increased demand for resources that exceed the environmental capacity, resulting in environmental degradation and inhibiting GTFP [46].

(2) Industrial structure. It is defined as the percentage of secondary sector value added to real GDP. The industrial structure can reflect a regional economic structure and development pattern [44]. An increase in the share of the secondary sector may inhibit the increase in GTFP [47]. In general, the larger the secondary industry scale, the worse the pollution in the area [48].

(3) Transportation level. Measured by per capita freight. Transportation can promote GTFP. On one hand, convenient transportation networks can lower transportation costs, improve transaction efficiency, as well as optimize resources allocation, which can lead to improved economic efficiency and GTFP growth [49]. On the other hand, the high transportation level can facilitate the sharing of environmental infrastructure, which in turn increases GTFP [50].

(4) Fiscal intervention. It is defined as the percentage of local budgetary expenditure to budgetary revenue. Fiscal intervention can reflect the government’s intervention on the economy and is an essential factor affecting GTFP [49]. When the government intervenes in economic activities in a limited way, it can alleviate the problem of market failure and reduce environmental pollution, which is beneficial to GTFP growth [51]. However, when the government intervenes excessively, it will lead to the disorder of the market and reduce the efficiency of resource allocation, which will inhibit GTFP [52].

(5) Foreign direct investment (FDI). It is described as the percentage of FDI in GDP. FDI is a double-edged sword for the development of GTFP. In one respect, the development of technological incentives from FDI can help stimulate the technical creativity of local enterprises and promote GTFP [53]. In another context, FDI also has the possibility to bring about environmental pollution, thereby inhibiting local GTFP [54].

Table 1 displays the detailed descriptions of the mentioned variables. We assemble the data from China Urban Statistical Yearbook (2003–2018), Provincial Statistical Yearbook (2003–2018), China Stock Market & Accounting Research (CSMAR) Database (2003–2018), and the official website portal of the Ministry of Ecology and Environment of the People’s Republic of China. All variables are based on data at the city level.

## 5. Empirical Estimation Results

In this part, first, we show the EPI’s average and dynamic treatment effects on GTFP; then, we run some robustness tests; and finally, we present the heterogeneity effects of the EPI on GTFP across cities with various initial GTFP levels and economic levels.

### 5.1. Average Treatment Effect of the EPI on GTFP

Table 2 first presents the evaluated findings of the EPI’s average treatment effects on GTFP based on the DID model. It shows the effect of the EPI on GTFP without and with control variables, respectively. We can find that the coefficient of the EPI is significant at 5% level and the result is robust, meaning that EPI can significantly promote GTFP.

Except directly use the DID method to analyze the GTFP differences between EPI cities and non-EPI cities. However, interviewed and non-interviewed cities possibly have systematic differences prior to EPI implementation, which may endanger our benchmark results. Hence, we adopt the propensity score matching and the difference-in-differences (PSM-DID) method to further estimate the average treatment effects of the EPI on GTFP. When applying the PSM-DID method, first, we employ the logit regression to value scores, where the EPI dummy is the explanatory variable, and the five control variables shown in Table 1 are taken as covariates. Second, we adopt radius matching, kernel matching, and nearest-neighbor matching to test whether the benchmark results are robust. Samples that failed to match the treatment group are excluded. Table 2 also reports the policy performance of the EPI assessed by PSM-DID. It documents that EPI’s coefficients are still significantly positive, which matches the DID results. Therefore, we conclude that EPI can boost GTFP in China, which validates our Hypothesis 1.

The likely causes of the EPI’s positive impact on GTFP are the following. First, the local government will enhance supervision over the enterprises’ production activities after EPI due to the pressure of the central government and public opinion [24]. The increased supervision of enterprises will decrease environmental pollution and benefit GTFP growth. Second, under the pressure of the EPI, enterprises will stimulate their green innovation ability to avoid being fined and thus promote GTFP [23].

### 5.2. Dynamic Treatment Effect of the EPI on GTFP

The previous benchmark results report the average effects of the EPI on GTFP from a static view. However, it is not clear whether EPI can have an impact on GTFP in the long run. Previous studies argued that the effect of the EPI can only last for a short period since only temporary measures can be taken to combat pollution in EPI [20]. Hence, it is inevitable to further investigate the dynamic impacts of the EPI on GTFP, thus providing more considerable significance for the optimization of the EPI in the future. Based on the model (3), we capture the dynamic effects of the EPI on GTFP, and Table 3 presents the dynamic estimation results without and with control variables, respectively.

Table 3 indicates that the EPI’s promotion effect on GTFP is not sustainable in the long term. Specifically, GTFP shows not an obvious improvement during the current year of the EPI but increased slightly in the first year after EPI realization, and this positive policy impact reached the maximum value in the second year of the EPI implementation. However, this promotion effect is not sustained in the third and fourth years after the implementation of the EPI. This suggests that EPI cannot maintain the long-term promotion effect on GTFP.

The likely causes of this result are the following. First, it takes a period for the effect of the EPI on GTFP to emerge, that environmental compliance supervision will have a regulatory and deterrent force on the area being interviewed in the year of the EPI, but this effect needs to be given a certain amount of time for the enterprises to respond [55]. Second, environmental governance measures of the EPI are compulsory and blunt, which are detrimental to the long-term development of GTFP. As a campaign-style environmental tool, EPI uses simple, brutal, and temporary measures, such as compulsorily sealing some polluting industries, to achieve short-term emission reduction tasks to improve the efficiency of environmental management [4]. However, these measures are detrimental to enterprises’ output increase, leading the long-term policy effect of the EPI on GTFP to be unsustainable [14]. Third, lacking long-term supervision will also impede the effectiveness of the EPI on GTFP. Under EPI, most local governments can achieve short-term pollution reduction targets which will hamper the public’s long-term supervision of the EPI. That explained the public no longer supervises the environmental situation because of rapid environmental improvements and a lack of incentives for enterprises to invest in environment for long-term improvements in environmental performance without public pressure [14].

### 5.3. Robustness Tests

The previous findings prove that the EPI can significantly promote GTFP. During this part, we perform additionally a train of tests to justify the previous practical results’ robustness, which includes a common trend test, a placebo test, excluding other policy’s impact, and winsorzing extreme value.

#### 5.3.1. Common Trend Test

The essential premise of applying the DID approach to the policy effect evaluation is to fulfill the common trend. To be specific, if there is no impact of the EPI, the GTFP of the treatment and the control groups are expected to acquire the identical trend. Therefore, referring to the method of Li et al. [56], this paper employs the event method to verify whether the research samples satisfied the common trend. The formula is shown below:

(8) $${GTFP}_{it}=\pi_{0}+\sum_{k\geq -4}^{k=4}\beta_{k}{EPI}_{it}^{k}+\beta_{5}X_{it}+\delta_{i}+\theta_{t}+\varepsilon_{it}$$

In detail, we assume the present year of policy implementation (${EPI}_{it}^{0}$) is the base period. ${EPI}_{it}^{k}$ represents the span within the EPI before and after implementation. ${EPI}_{it}^{k<0}=1$ represents the prior-EPI city. ${EPI}_{it}^{k\geq 0}=1$ denotes the post-EPI city. The coefficients for ${EPI}_{it}^{k<0}$ should be paid attention to. If the coefficients for ${EPI}_{it}^{k<0}$ does not pass the significance test, which suggests the common trend hypothesis is fulfilled.

**Figure 3:**
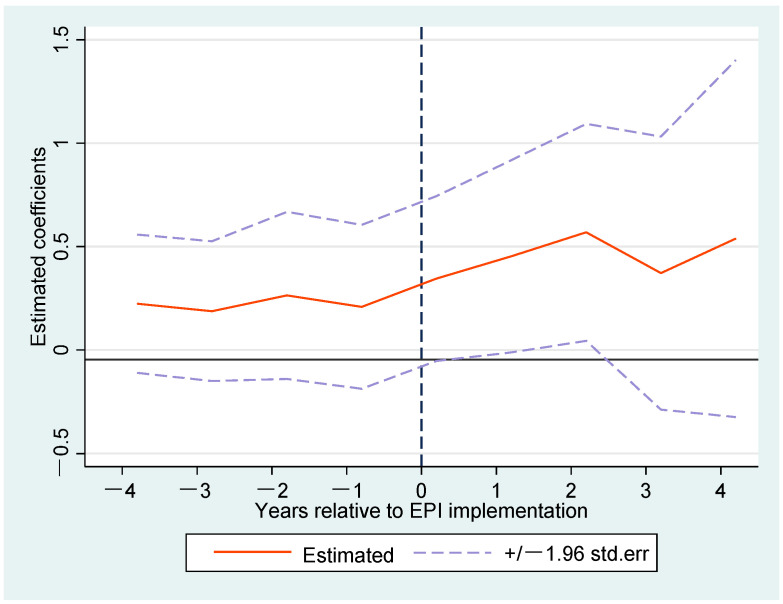
Common trend test.

Figure 3 shows common trend test findings where the assumption is fulfilled. In contrast to the base period (${EPI}^{0}$), the coefficients for all ${EPI}^{k<0}$ oscillate around zero and do not pass the significance test, suggesting that the coefficients of ${EPI}^{k<0}$ do not have any significant difference with the base period ${EPI}^{0}$. Thus, we conclude that it is accurate for the common trend test, and the benchmark findings are reliable.

#### 5.3.2. Placebo Test

To overcome the chance that EPI’s effect on GTFP is affected by omitted variables, we followed the practice of Beck et al. [34] to exercise a virtual experimental group and control group for the placebo test. Specifically, we first generate a false list of the EPI cities by random sampling cities implementing EPI, then we rerun the DID method for policy effect estimation based on the generated false EPI variables. If the estimates of false EPI variables are not significant, then there are no other omitted variables influencing the estimates. To assure the test’s robustness, we repeat the procedure on 1000 occasions.

**Figure 4:**
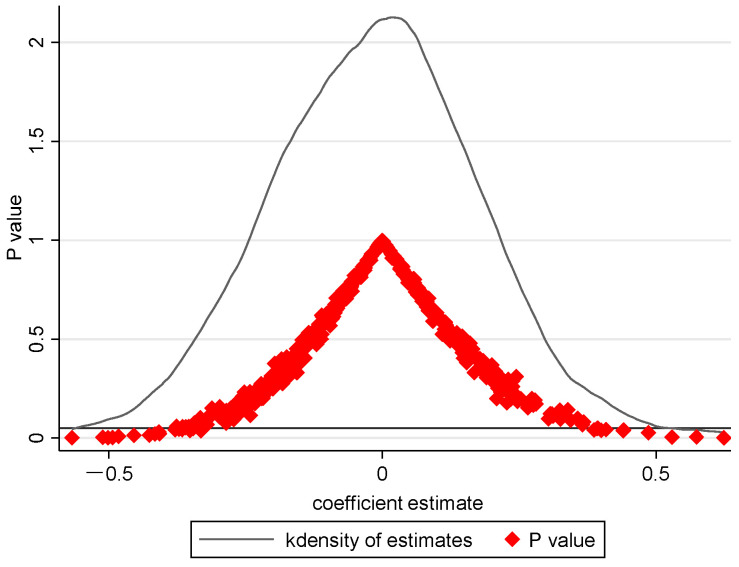
Placebo test.

Figure 4 reports the spread of estimated factors and *p*-values for 1000 random samples. It presents that the estimated factors are mostly distributed around 0 and the *p*-values are mostly distributed above the 10% level, which is different from the baseline results. Therefore, EPI’s impact on GTFP is unlikely to be influenced by other omitted variables, which reinforces the robustness of our benchmark results.

#### 5.3.3. Eliminating the Interference of Other Policies

Concerning that, there are probably more policies associated with the GTFP during EPI realization, and EPI’s effect on GTFP may have been influenced. To deal with this problem, it is necessary to eliminate the disturbance of other policies to carry out robustness tests. Based on this, this study collects two policies that might affect GTFP in the same period, including the “Eco-civilization pilot demonstration zone” and the “Low-carbon pilot”.

First, the “Ecological civilization pilot demonstration zones policy” can be considered an interference policy. This policy is implemented under the National Development and Reform Commission in December 2013. As of 2018, more than 100 cities (regions) have been selected as ecological civilization demonstration zones. Those demonstration zones aim to promote green growth through the restructuring industries, upgrading industrial structures, and accelerating green production and ecological systems [57]. Therefore, this policy may have an impact on GTFP during the study period.

Second, “Low-carbon pilot” should be considered as the second policy. To decrease carbon emissions and increase green growth, the National Development and Reform Commission (NDRC) initiated the 1st group of low-carbon city pilots in 2010 and instituted the 2nd and 3rd groups of low-carbon city pilots in 2012 and 2017, respectively. Until 2018, a total of 81 cities have been listed as low-carbon pilot cities. The establishment of low-carbon pilot cities can enhance resource allocation and promote the efficiency of resource consumption. Besides, it can also drive scientific and technical creativity, boost the industrial structure upgrading, and ultimately realize an increase in GTFP [50]. For example, Yu and Zhang [58] demonstrates that a low-carbon pilot policy can improve carbon emission efficiency. Thus, low-carbon city pilot policies are the potential to have an influence on GTFP during the study period.

Following the study of Li et al. [56], our study controlled for dummy variables to distinguish whether the sample cities were ecological civilization demonstration zones or low-carbon pilot cities in the DID benchmark regression. This was done to accurately measure the effect of the EPI without the interference from the aforementioned policies. If the regression result is generally matching the baseline result, it implies that the implementation of other policies did not impede the policy impact of the EPI.

Table 4 provides evidence that the EPI’s significant positive effect on GTFP remains intact after the addition of dummy variables for two policies. This result is comparable to the baseline, which suggest that the implementation of other policies does not interfere with the EPI’s impact, which provide further support for the robustness of the baseline regression.

#### 5.3.4. Winsorzing Extreme Value

To decline the impact of the extremum on our results, we further winsorize the sample at the level of 5% and 10% to eliminate the extremum and check the robustness of our results. Table 4 presents the research consequences after removing outliers. EPI’s impact on GTFP does not change dramatically compared with the baseline findings, and the findings remain statistically significant, which further reinforcing the robustness of our results.

### 5.4. Heterogeneity Analysis

In this section, we investigate whether EPI’s effect on GTFP varies among cities with various initial GTFP levels and economic development levels.

#### 5.4.1. Heterogeneity Analysis with Different Initial GTFP Levels

Guo et al. [59] argues that the initial level of GTFP may affect the EPI’s effect. Thus, we put our sample cities into two groups on account of the median of GTFP: one group comprised of cities with low initial GTFP levels, and the other of cities with high initial GTFP levels. Table 5 presents the heterogeneity results across different initial GTFP levels.

Table 5 demonstrates that EPI has a significant positive effect on the cities with low initial GTFP levels, whereas in those cities with high GTFP levels the effect of the EPI on GTFP is not significant. The likely causes of this result are the following: first, cities with low initial GTFP often have backward technologies and inefficient resources to promote GTFP [60]. The implementation of the EPI can induce these cities to upgrade their production technologies and improve resource utilization efficiency, which is beneficial to GTFP growth. While for cities with high initial GTFP, the improvement space of GTFP is relatively limited [59]. Second, cities with high initial GTFP levels already have more advanced green technologies, thus there is relatively limited potential for improvement in GTFP [12].

#### 5.4.2. Heterogeneity Analysis across Different Economic Development Levels

The impact of the EPI will also be influenced by the economic development level. For instance, Pang et al. [61] suggested the effectiveness of enforcement environment legislation is contingent upon the degree of economic growth within a certain range. Furthermore, Liang and Yang [62] also identified that the effect of environmental legislation on eco-efficiency is varied across regions with different economic levels. Therefore, it is essential to evaluate the heterogeneity effects across various economic growth levels. We put the sample cities into economically developed areas and economically underdeveloped areas according to the median per capita GDP and conducted regression analysis, respectively.

Table 5 reveals the results that EPI’s influence on GTFP is significant at a low economic level, as evidenced by the coefficient of the EPI in underdeveloped areas is 0.423, which is higher than the policy effect in baseline regression (0.356) This effect, however, is not seen in more developed areas, with the coefficient of the EPI not being significant.

The likely causes of this result are the following: First, underdeveloped areas are always dominated by traditional industries with inefficient production modes, high resource consumption and environmental pollution, and low green development level [63]. The enforcement of the EPI will lower these areas’ environmental pollution and increase their economic level, thus can promote GTFP. Second, the GTFP level is already relatively high in developed areas due to advanced production technologies and government financial support [64]. Therefore, the improvement potential of GTFP is relatively limited in these developed areas and the impact of the EPI is relatively weak [65].

## 6. Mechanism Analysis

As the theoretical framework discussed in Section 3, EPI may affect GTFP through technical creativity and industrial structure upgrading. In this section, we empirically test these potential impact mechanisms. We construct a mediating effects model to validate both of these influential mechanisms according to Tofighi and MacKinnon [66]:

(9) $${GTFP}_{it}=\alpha_{0}+\alpha_{1}{EPI}_{it}+\alpha_{2}X_{it}+\delta_{i}+\theta_{t}+\varepsilon_{it}$$

(10) $${Mediation}_{it}=\omega_{0}+\omega_{1}EPI+\omega_{2}X_{it}+\mu_{i}+\gamma_{t}+\varepsilon_{it}$$

(11) $${GTFP}_{it}=\gamma_{0}+\gamma_{1}EPI+\gamma_{2}{Mediation}_{it}+\gamma_{3}X_{it}+\mu_{i}+\gamma_{t}+\varepsilon_{it}$$

where ${Mediation}_{it}$ represents the two mediation variables: technical creativity and industrial structure upgrading. Regarding technical creativity, it is determined as the percentage of green patents to the total patent grants [63]. In terms of upgrading industries, we employ the percentage of the tertiary industry to secondary industry to measure it [67]; $\alpha_{1}$ denotes the total effect of ${EPI}_{it}$ on ${GTFP}_{it}$; $\gamma_{1}$ denotes the direct effect of ${EPI}_{it}$ on ${GTFP}_{it}$ and $\omega_{1}\times \gamma_{2}$ represents indirect effect of ${EPI}_{it}$ on ${GTFP}_{it}$. Other variables’ definition is matched with Equation (1).

The following is a brief description of the mediation effect model process. First, we inspect whether $\omega_{1}$ and $\gamma_{2}$ are significant, which means not equal to 0. If the two coefficients are significant, and $\omega_{1}\times \gamma_{2}$ and $\gamma_{1}$ have the identical symbols, the mediating effect is affirmatory. Second, if either $\omega_{1}$ or $\gamma_{2}$ is not significant, we will further run a Bootstrap test to check whether the mediating effect is set up. If the result passes the significance test and $\omega_{1}\times \gamma_{2}$ has the identical symbol as $\gamma_{1}$, the mediating effect is affirmatory, too.

Table 6 documents the impact of the EPI on GTFP and the mechanism test results of technical creativity. The signs of the coefficients of the EPI $\omega_{1}$ (0.183) and technical creativity $\gamma_{2}$ (0.006) are in harmony with our expectations but failed the significance test. Thus, we applied the Bootstrap test to check whether the mediating effect exists, and Table 7 demonstrates the results. The results show that $\omega_{1}\times \gamma_{2}$ is 0.137 and significant at the level of 10%, proving that technical creativity is a mechanism. Our results are consistent with the findings of Wang and Yue [68] and Lv et al. [13], who also find that EPI has a significant positive impact on the green innovation of enterprises. Facing the increasing pressure of the EPI, the enterprises in EPI cities choose either reduce production or increase technological innovation. However, the choice of reducing production conflicts with the enterprises’ needs of maximizing profits and maintaining competitiveness. And the continuous increase of end-of-line management costs serves to further incentivize enterprises to undertake technological innovation. Therefore, the greater pressure of environmental regulation faced by enterprises, the more inclined they are to carry out technological innovation.

Table 6 reports the mechanism test findings of industrial structure upgrading. We discover that the coefficient of industrial structure upgrading $\gamma_{2}$ (0.461) is significant, and $\omega_{1}\times \gamma_{2}$ has a positive sign, which is consistent with the coefficients of the EPI $\gamma_{1}$ (0.332). The above results indicate that industrial structure is one of the mechanisms to advance the GTFP. Under the pressure of the EPI, many high-polluted industries are forced to shut down and environmentally friendly industries are welcomed by the local governments; thus, the industrial structure is upgraded to a cleaner direction and can promote GTFP. For example, after the EPI, 57 polluted companies were shut down in the Linyi city of Shandong Province and 500 smelting enterprises were closed in the Hengyang city of Hunan Province.

## 7. Conclusions and Policy Implementation

As a large-scale transition and the most stringent environmental monitoring program in Chinese history, the effectiveness of the EPI has long been a subject of interest to researchers. Numerous studies have investigated the environmental performance of the EPI, and they have reached a consistent conclusion that the EPI can reduce environmental pollution. However, few studies have empirically analyzed the economic performance of the EPI. Additionally, their arguments are controversial, which hinders our understanding of the true economic performance of the EPI. To fill this void, this study regarded the EPI as a quasi-natural experiment and employed a DID method to quantify the EPI’s effectiveness on GTFP. We acquired the following results: first, the EPI can have a promotion influence on GTFP, but the influence is not sustainable in the long run. A series of robustness probes validated that this finding is robust and credible. Second, a heterogeneity analysis revealed that the impact of the EPI on GTFP is greater in cities that demonstrate low initial GTFP performance and low economic performance. Third, further exploration of the mechanism shows that technical creativity and industrial structure upgrading are the two main mechanisms through which the EPI can have a promotional influence on GTFP.

In the light of these conclusions, we would like to propose the following recommendations.

First, the central government should continue to promote the implementation of the EPI, thus promoting GTFP growth. Our analysis shows that the EPI can achieve the dual effect of pollution control and green development, indicating that the EPI has achieved great success in monitoring local government behavior. Therefore, to ensure the long-term effectiveness of the EPI, China should further strengthen the supervision and feedback mechanism of the EPI. This can be accomplished through several measures: requiring the interviewed local governments to provide regular feedback regarding issues related to the EPI; establishing an internal–external joint supervision model, such as incorporating the public and media to monitor the implementation of environmental policy in the interviewed places; applying a regular feedback assessment system for interviewed areas; and introducing a third-party assessment mechanism.

Second, local governments should optimize enterprises’ innovation environment and accelerate industrial structure upgrading to boost GTFP. Our analysis shows that technical creativity and industrial structure upgrading are two major mechanisms for the EPI to promote the GTFP. Therefore, local governments should take effective measures toward these two mechanism paths, ensuring the long-term effect of the EPI. For example, the central government can provide a relaxed technical creativity environment for the interviewed areas, inducing the enterprises to focus more on fostering new industries that are conducive to technical creativity and clean energy development. In addition, the central government can also provide local governments with corresponding environmental subsidies to help them upgrade their industrial structure, thus promoting GTFP.

Although this research provided some valuable findings and enlightenment for the government’s decision-making and research in the field of environmental regulation and green economic growth, some limitations should be noted. First, our research only took China’s EPI into account. No cases of other emerging-market countries were introduced, and there is a lack of more extensive identification verification. Second, due to the limited number of years of observation, we only explored the effect of the EPI on GTFP from 2003 to 2018. Future research can complement and extend the policy effects of the EPI in terms of timeliness by adjusting the research methodology and research design.

## Figures and Tables

**Table 1:** Definition and description of variables.

Main Variables	Description	Mean	SD
GTFP	GML index calculated based on the directivity distance function method	1.66	1.42
EPI	=1, If the city implemented EPI in year t; =0, otherwise	0.04	0.18
Population density	The total population as a percentage of administrative area (people/km^2^)	435.65	330.79
Industrial structure	The percentage of secondary sector value added to real GDP	47.92	10.71
Transportation level	per capita Freight (ton/person)	44.91	163.08
Fiscal intervention	The percentage of local budgetary expenditure to budgetary revenue (%)	2.57	1.80
Foreign direct investment	The percentage of foreign direct investment in GDP (%)	0.02	0.02

**Table 2:** Average treatment effect of the EPI on GTFP.

Variables	DID			PSM-DID		
				Radius Matching	Kernel Matching	Nearest-Neighbor Matching
EPI	0.289 * (0.166)	0.356 ** (0.173)	0.356 ** (0.161)	0.402 ** (0.183)	0.350 * (0.179)	0.400 ** (0.183)
Control variables	No	Yes	Yes	Yes	Yes	Yes
Year fixed effects	Yes	Yes	Yes	Yes	Yes	Yes
City fixed effects	Yes	Yes	Yes	Yes	Yes	Yes
R^2^	0.412	0.434	0.434	0.706	0.699	0.706
Observations	4272	4068	4068	3119	4045	3119

Note: Robust standard errors clustered at the city level are reported in parentheses except the Column (3)’s standard errors clustered at the province level; (2) * and ** are significant at 10% and 5%, respectively.

**Table 3:** Dynamic treatment effect of the EPI on GTFP.

Variables	Dynamic Treatment Effect of the EPI on GTFP	
EPI^0^	0.215 (0.158)	0.246 (0.157)
EPI^1^	0.318 * (0.185)	0.389 * (0.194)
EPI^2^	0.430 * (0.257)	0.464 * (0.270)
EPI^3^	0.233 (0.289)	0.409 (0.289)
EPI^4^	0.394 (0.445)	0.695 (0.475)
Control variables	No	Yes
Year fixed effects	Yes	Yes
City fixed effects	Yes	Yes
R^2^	0.412	0.437
Observations	4272	4066

Note: (1) Robust standard errors clustered at the city level are reported in parentheses, the same as below. (2) * is significant at 10%.

**Table 4:** Eliminate the interference of other policies and winsorzing extreme value.

Variables	Eliminate the Interference of the Policy of Ecological Civilization Pilot	Eliminate the Interference of Low-Carbon City Pilot Policy	Winsorzing Extreme Value (5%)	Winsorzing Extreme Value (10%)
	0.344 * (0.181)	0.384 ** (0.178)	0.356 ** (0.173)	0.255 ** (0.121)
EPI	Yes			
Ecological civilization pilot city dummy variable		Yes		
Low carbon pilot city dummy variable	Yes	Yes	Yes	Yes
Control variables	Yes	Yes	Yes	Yes
Year fixed effects	Yes	Yes	Yes	Yes
City fixed effects	0.700	0.701	0.434	0.358
R^2^	4068	4068	4068	3269
Observations	0.344 * (0.181)	0.384 ** (0.178)	0.356 ** (0.173)	0.255 ** (0.121)

Note: * and ** are significant at 10% and 5%, respectively.

**Table 5:** Heterogeneity results across different initial GTFP levels and economic development levels.

Variables	High Initial GTFP Level	Low Initial GTFP Level	High Economic Level	Low Economic Level
EPI	0.038 (0.212)	0.233 ** (0.139)	0.372 (0.251)	0.423 * (0.250)
Control variables	Yes	Yes	Yes	Yes
Year fixed effects	Yes	Yes	Yes	Yes
City fixed effects	Yes	Yes	Yes	Yes
R^2^	0.766	0.558	0.701	0.702
Observations	2004	2064	2052	2016

Note: * and ** are significant at 10% and 5%, respectively.

**Table 6:** Eliminating the interference of other policies and winsorzing extreme values.

Variables		Technical Creativity Effect		Industrial Structure Upgrading Effect	
	GTFP	Technical Creativity	GTFP	Industrial Structure	GTFP
EPI	0.356 ** (0.173)	0.183 (0.211)	0.325 * (0.172)	0.053 ** (0.023)	0.332 * (0.172)
Technical creativity			0.006 (0.051)		
Industrial structure					0.461 ** (0.234)
Constant term	4.221 *** (0.400)	0.983 (1.025)	4.302 *** (0.406)	2.666 ** (0.087)	2.992 *** (0.726)
Control variables	Yes	Yes	Yes	Yes	Yes
Year fixed effects	Yes	Yes	Yes	Yes	Yes
City fixed effects	Yes	Yes	Yes	Yes	Yes
R^2^	0.434	0.478	0.442	0.768	0.438
Observations	4068	3960	3960	4068	4068

Note: *, **, and *** are significant at 10%, 5% and 1%, respectively.

**Table 7:** Test of the technical creativity effect of the EPI on GTFP.

Bootstrap Test	Technical Creativity	SE	*p*-Value
Indirect effect	0.137	(0.082)	0.095

## Data Availability

Data supporting this study are included within the article.
